# A case of biliary cystic tumor with repeated hemobilia

**DOI:** 10.1186/s40792-014-0006-0

**Published:** 2015-04-08

**Authors:** Gen Takahashi, Shintaro Kuroda, Hirotaka Tashiro, Tsuyoshi Kobayashi, Kohei Ishiyama, Kentaro Ide, Hiroyuki Tahara, Masahiro Ohira, Koji Arihiro, Hideki Ohdan

**Affiliations:** Department of Gastroenterological and Transplant Surgery, Hiroshima University Hospital, 1-2-3, Kasumi, Hiroshima, 734-8551 Japan; Department of Anatomical Pathology, Hiroshima University Hospital, 1-2-3, Kasumi, Hiroshima, 734-8551 Japan

**Keywords:** Hemobilia, Biliary cystic tumor, IPNB

## Abstract

Intraductal papillary neoplasm of the bile duct (IPNB) is classified as a biliary cystic tumor with a tendency of causing obstruction. Neoplastic cases involving hemobilia are rarely reported. We herein describe a case of biliary cystic tumor with repeated hemobilia. A 57-year-old woman was histologically diagnosed with cavernous hemangioma. During the follow-up period after transcatheter arterial embolization (TAE), she experienced repeated hemobilia, and multiple other TAE sessions were performed for hemostasis. She was referred to our hospital 8 years after the first surgery owing to a growing tumor. Histopathological examination after extended right hepatectomy and caudate lobectomy indicated IPNB with an associated invasive carcinoma. Six months thereafter, computed tomography revealed a recurrent liver tumor and a nodule in the abdominal cavity. She died 36 months after the second surgery, despite chemotherapy. Our experience suggests that IPNB should be considered during differential diagnosis of dilated hepatobiliary tumors with hemobilia.

## Background

Hemobilia is usually caused by trauma, iatrogenic events, cholangitis, and cholelithiasis, but rarely associated with a tumor [1]. On the other hand, biliary cystic tumors are rare neoplasms arising in the liver and extrahepatic biliary system at a lower frequency [2]. The latest World Health Organization (WHO) Classification of Tumors of the Digestive System published in 2010 codified and defined hepatic mucinous cystic neoplasms (MCN) and intraductal papillary neoplasms of the bile duct (IPNB) as the counterparts of pancreatic MCN and intraductal papillary mucinous neoplasm (IPMN-P) [3]. IPNB is identified as a premalignant lesion or intraepithelial neoplasm of the bile duct, and it is relatively rare among all bile duct cancers. Here, we report a case of biliary cystic tumor with repeated hemobilia that was followed up for 8 years. We also present its histological characteristics and discuss relevant literature.

## Case presentation

A 57-year-old woman was previously admitted to a hospital because of abdominal pain. Gastrointestinal fiberscopy showed hemobilia, whereas abdominal ultrasonography and computed tomography (CT) performed at the time showed two tumors in the liver: one in the lateral segment measuring 4 × 3 cm in diameter and the other in the posterior segment measuring 10 × 5 cm in diameter. CT showed no clear differences between the two tumors; they were multiple capsulated lesions filled with cystic contents and highly enhanced mural nodules (data not shown). Additionally, the needle biopsy for both tumors did not reveal a malignant finding - a fragment of liver tissue with no malignancy. Despite the tumors being thought as benign, she underwent a diagnostic partial resection of the lateral segment. Histological examination indicated cavernous hemangioma. She then received transcatheter arterial embolization (TAE) for hemostasis of the right lobe tumor. During the follow-up period thereafter, she experienced six episodes of hemobilia, and four other TAE sessions were performed. She was referred to our hospital 8 years after the diagnostic surgery owing to the growing tumor in the right lobe.

The patient's medical history included an appendectomy at 9 years of age, Cesarean section at the age of 26 and 29 years, and right lower lobectomy with partial resection of the lungs' left upper lobe for adenocarcinomas at the age of 53 years. She had no history of liver disease, including hepatolithiasis.

Laboratory results on admission were as follows: hemoglobin, 11.1 g/dL; total bilirubin, 0.5 mg/dL; aspartate aminotransferase, 65 IU/L; alanine aminotransferase, 54 IU/L; alkaline phosphatase, 819 IU/L; and γ-glutamyl transpeptidase, 227 IU/L. Levels of alpha-fetoprotein and protein induced by vitamin K absence were within normal limits, while those of carcinoembryonic antigen and carbohydrate antigen 19–9 were elevated to 12.8 ng/mL and 862 U/mL, respectively. Viral markers for hepatitis B and C were negative.

**Figure 1:**
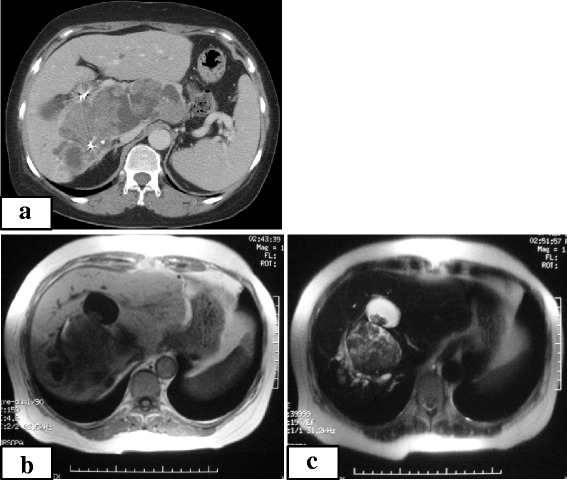
**Abdominal contrast computed tomography (post-transcatheter arterial embolization [TAE]) and magnetic resonance imaging findings. (a)** Computed tomography scan showed a low-density hepatic lesion measuring 15 cm in diameter with mural nodules. These nodules were contrast-enhanced. The TAE coils were observed. **(b)** T1-weighted image showed a low-intensity mass, except for the mural nodules. **(c)** T2-weighted image demonstrated high signals from the mural nodules.

**Figure 2:**
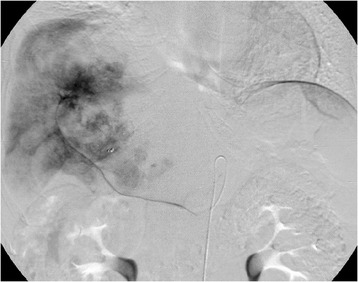
**Transcatheter arterial embolization sequence as shown in a right hepatic angiography.** The angiography also demonstrated hypervascular lesions in the cystic tumor.

On physical examination of the abdomen, the liver edge was palpable four fingerbreadths beneath the xiphoid process. Abdominal ultrasonography revealed a multilocular oval mass with papillary infolding measuring 10 × 15 cm located in the right hepatic lobe. CT scans showed a low-density hepatic tumor measuring 15 cm in diameter that occupied almost the entire right and caudal lobes, compressing the portal vein. The nodules were contrast-enhanced (Figure 1a). T1-weighted magnetic resonance imaging showed a low-intensity mass, except for the mural nodules. The signals from these mural nodules were high on T2-weighted images (Figure 1b,c). A superior mesenteric arteriography showed the right hepatic artery originating from the superior mesenteric artery with the tumor stain present (Figure 2). Fluorodeoxyglucose positron emission tomography/CT showed high tumor uptake.

**Figure 3:**
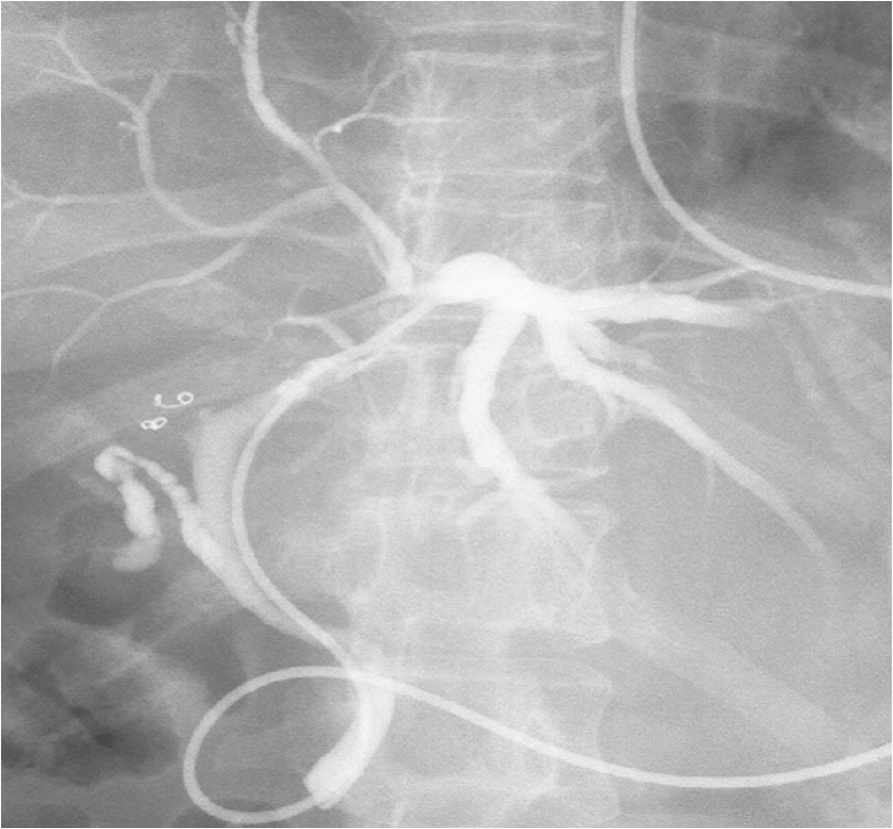
**Endoscopic retrograde cholangiography findings.** Endoscopic retrograde cholangiography showed obstruction of the right hepatic bile duct, compression of the left hepatic duct, and dilatation of the peripheral bile ducts.

Endoscopic retrograde cholangiography demonstrated obstruction of the right hepatic bile duct, compression of the left hepatic duct, and dilatation of the peripheral bile ducts (Figure 3). The cystic lesion was suspected as a mucin-producing liver tumor, such as MCN of the liver or IPNB. Biliary cytology was diagnosed as papillary adenocarcinoma *in situ* or papillary adenoma with severe atypia.

**Figure 4:**
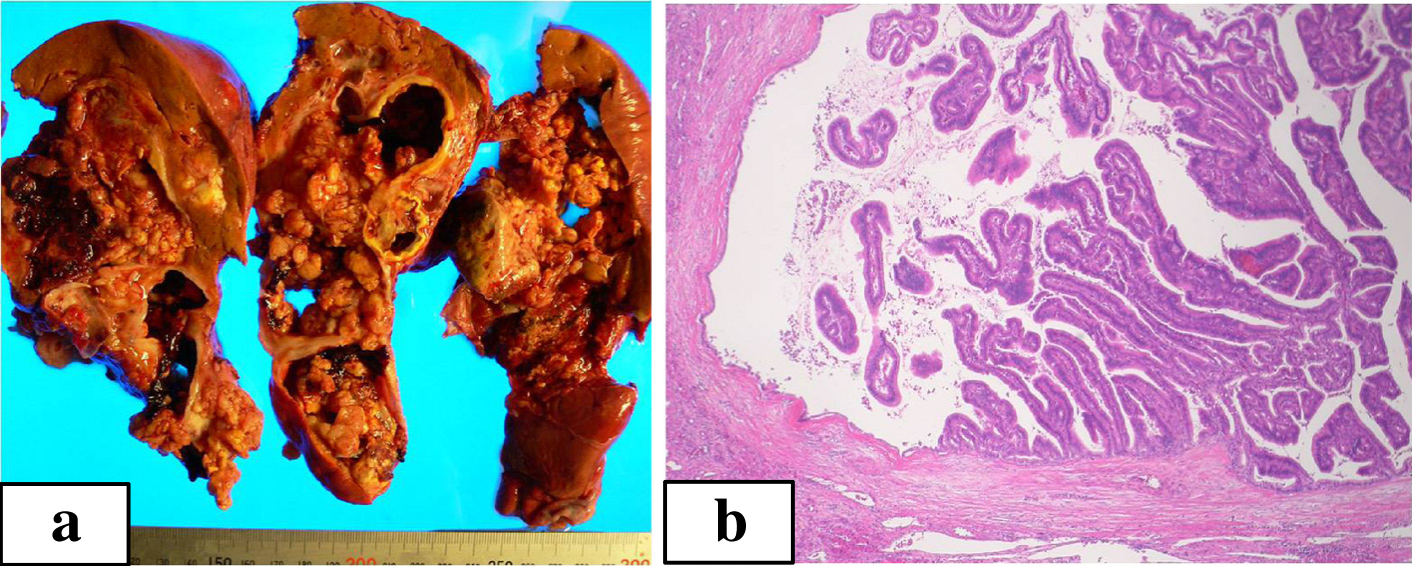
**Macroscopic and histopathological findings from the resected specimens. (a)** The cut surface of the resected specimen showed a multilocular cystic lesion with many papillary nodules projecting from the lining wall, as well as transparent mucinous fluid. **(b)** The tumor was histologically composed of a well-differentiated adenocarcinoma with papillary structures. The cyst wall was lined with thick hyalinized connective tissue, and stromal invasion of the tumor was observed (hematoxylin and eosin stain, ×100).

The patient underwent right hemihepatectomy (stipulate + Sg1) with cholecystectomy and concurrent splenectomy owing to the growing aneurysm of the splenic artery. Macroscopic observation indicated a direct connection between the cystic lesion and the right hepatic bile duct. During the operation, we ligated bile ducts and gave attention not to spill bile into the abdominal cavity. The resected multilocular tumor was filled with clear mucus, and the cut surface showed multiple yellowish papillary nodules lining the cystic wall. The cyst had a direct luminal communication with the right hepatic duct. Histological examination indicated the biliary cystic lesion was a well-differentiated adenocarcinoma (Figure 4). Ovarian-like stroma was not evident. Thus, the final diagnosis was IPNB with an associated invasive carcinoma. The patient was discharged 51 days after surgery, despite experiencing cholangitis and enteritis previously during the post-operative course.

Six months after surgery, CT scans revealed a recurrent liver tumor and a nodule in the abdominal cavity. Unfortunately, the patient died 36 months after surgery, despite chemotherapy.

### Discussion

Hemobilia was first reported by Sandblom in 1948. It is a rare condition often caused by trauma, iatrogenic events, cholangitis, or cholelithiasis [1], but rarely by neoplasms (5% to 6% of all cases) [4]. Its symptom is known as the trilogy of Quincke, upper gastrointestinal hemorrhage, epigastralgia, and jaundice. When the biliary tract is obstructed by blood clots, abdominal pain and jaundice occur because of increased pressure in the tract. In the presented case, our patient experienced abdominal pain, but not jaundice, and her laboratory results indicated anemia with abnormal liver function.

Angiography is recommended for the examination and treatment of uncontrolled hemobilia [5]. If hemorrhage occurs from an artery, TAE is the first choice of treatment and often effective. Although our patient was successfully treated with TAE for the six hemobilia episodes during her 8-year hemangioma follow-up, hemorrhage recurred frequently, and the tumor was growing. She therefore underwent surgical intervention and was finally diagnosed with IPNB and an associated invasive carcinoma.

Common symptoms and complications of IPNB are abdominal pain, liver function disorder, jaundice, or fever caused by the bile duct obstruction due to hypersecretion of mucin or the tumor itself [6,7]. We could not find a previous report of hemobilia caused by IPNB by searching MEDLINE using the English language. Sugawara et al. suggested that the risk of hemobilia from neoplasms depended on tumor size and histological type, especially in cases of soft and fragile papillary tumors [8]. Biliary cystic tumors are rare neoplasms occurring in the liver and, less frequently, the extrahepatic biliary system [2]. They account for less than 5% of all reported cystic lesions of the liver [9].

According to the 2010 WHO Classification of Tumors of the Digestive System, biliary cystic tumors are no longer defined as biliary cystadenoma, papillomatosis, papillary adenocarcinoma, or biliary cystadenocarcinoma, but instead are redefined as IPNB and MCN of the liver [3]. IPNB is identified as a premalignant lesion or intraepithelial neoplasm of the bile duct and divided into intraductal papillary neoplasms with low-, intermediate-, or high-grade intraepithelial neoplasia, according to tumor cell grade [7,10]. Papillary tumors of the bile duct are relatively rare with a reported prevalence of 4% to 15% among all bile duct cancers [11-13]. On the other hand, MCN of the liver is the biliary counterpart of pancreatic MCN. The disease usually occurs in women and does not exhibit a luminal communication with the pancreatic or bile ducts [14]. MCN also differs from IPNB with respect to the expression of estrogen and progesterone receptors, owing to the presence of a subepithelial ovarian-like stroma in the former. In this case, the intrahepatic bile ducts were markedly dilated with papillary proliferation in the lumen, and the tumor cells exhibited marked atypical appearance. Ovarian-like stroma was not identified. Thus, our final diagnosis was IPNB with an associated invasive carcinoma.

Ogawa et al. reported several CT findings of IPNB [15], including the presence of intraductal mass, extensive infiltration along the bile duct, intense enhancement rim at the base of the mass, and isodense or hyperdense lesions relative to the normal hepatic parenchyma during the late arterial phase. In our case, the tumor was enhanced from the portal to the venous phase with obstruction of the right hepatic bile duct. Although hemangioma, which was our preoperative diagnosis, could also exhibit a tendency of volume increasing and contrast enhancement as a result of bleeding, the resected tumor in this case presented as a multilocular cyst filled with multiple yellowish papillary nodules, hypersecretion of clear mucin, and granular mucosa growing directly into the bile duct lumen. As also observed in IPMN-P, columnar and cuboidal tumor cells of IPNB have papillary or villous structures and show expansive growth into the lumen of the bile duct with fibrous vascular stroma.

The CT findings in our case showed well-enhanced massive papillary contents in the dilated bile ducts with extravasation. The intraductal papillary contents evident by contrast CT further indicated that the tumor was present in the bile ducts instead of the liver parenchyma. Moreover, the repeated hemobilia episodes were also indicative of the bile ducts' involvement.

IPNB are recognized as slow-growing neoplasms compared to normal intrahepatic bile duct carcinoma [16]. Several articles present and recommend radical surgical management as the first choice of treatment for IPNB [6,17], with a reported 5-year survival rate after radical surgery ranging from 31% to 80% [18]. But in a latest big study, Kubota et al. reported better prognosis of resected IPNB [7]. They emphasize the necessity of complete resection, and the 1-, 3-, 5-, and 10-year overall survival rates for the 105 mucin-producing IPNB patients were 96%, 90.4%, 84%, and 81.1%. If a recurrent tumor is present, radical surgical therapy must be planned owing to its slow growing characteristic [6].

In this case, only the lateral lesion was resected for diagnostic purposes in the initial surgery, owing to the lack of any malignant impression. The residual lesion in the right lobe showed slow progression, with repeated hemobilia. IPNB also exhibits slow clinical progression, despite the possibility of direct invasion to the bile duct, leading to its dilatation, jaundice, or hemobilia.

## Conclusions

Our case presented a thought-provoking hepatobiliary course. The patient was initially followed for a slow-growing hepatic hemangioma, but eventually diagnosed with IPNB. It was challenging to distinguish the two conditions. Furthermore, although there are several reports of hemobilia originating from hepatic hemangioma, it is very rare in IPNB. Nonetheless, our experience in this case suggests that IPNB should be considered during differential diagnosis of dilated hepatobiliary tumors with hemobilia.

## Consent

Witten informed consent was obtained from the patient for publication of this case report and any accompanying images.

## Abbreviations

CT: computed tomography
IPMN-P: intraductal papillary mucinous neoplasm of the pancreas
IPNB: intraductal papillary neoplasm of the bile duct
MCN: mucinous cystic neoplasm
TAE: transcatheter arterial embolization
WHO: World Health Organization
